# Persistent Neurological, Dissociative, and Amnestic Symptoms Following a Mild Traumatic Brain Injury in an Adolescent: A Complex Case of Conversion Disorder

**DOI:** 10.7759/cureus.42755

**Published:** 2023-07-31

**Authors:** Martin Leczycki, Douglas J Berne, Daisy V Shirk, Jerry M Sayers

**Affiliations:** 1 Psychiatry, Reading Hospital/Tower Health, West Reading, USA

**Keywords:** conversion disorder, conversion, amnesia, dissociation, dissociative amnesia, tbi, trauma

## Abstract

The diagnosis of conversion disorder may be given to patients with unexplained neurological symptoms after the exclusion of medical and organic etiologies, suggesting a psychiatric etiology. It requires a thorough examination of all contributing factors across the biopsychosocial model. With a variety of possible presentations, the evaluation and treatment of conversion disorder should reflect its complexity. This case report describes a case of conversion disorder complicated by mild traumatic brain injury and acute psychological re-traumatization in an adolescent with social anxiety and focuses on the connection between symptoms of conversion and dissociation with trauma and memory to form an understanding of the unique presentation and treatment of this condition.

## Introduction

Conversion disorder, also known as functional neurological symptom disorder, may be broadly defined as a psychiatric illness presenting with neurological symptoms that cannot be fully explained by a recognized neurological or medical condition, causing impairment in social, occupational, or other important areas of function. Symptoms disrupt motor, sensory, or cognitive function and may vary in severity and course but are generally believed to have an acute or subacute onset following an often-unknown stressor that can be physical or psychological in nature [1-3]. The term "conversion disorder" was coined by Sigmund Freud, who hypothesized that the occurrence of certain symptoms not explained by a medical condition reflects unconscious conflict, with the word "conversion" referring to the substitution of a somatic symptom for a repressed idea or unacceptable desire. As such, symptoms may represent an unconscious defense mechanism to cope with psychological conflict. The International Classification of Diseases classifies conversion disorder as a dissociative disorder, a condition involving disruption of memory, awareness, identity, or perception. Dissociative symptoms are often thought to be associated with trauma, though the effects of trauma on these processes are not fully understood [4,5]. When experiencing highly emotional or stressful events, physiological responses such as those involving the limbic system lead to more consolidated memories. Studies show that stress enhances explicit memory directly related to a stressful event [6,7]. On the other hand, some would argue for the existence of repressed memories, a concept rooted in psychoanalytic theory wherein repression is understood as a defense mechanism that excludes painful experiences or unacceptable desires from the conscious mind. The degree and nature of impairment to explicit and implicit memory due to trauma can vary, but it has been suggested that acute re-traumatization can bring repressed memories back to conscious awareness, causing internal conflict that may produce physical symptoms such as those seen in conversion disorder.

Describing the etiology and symptomatology of conversion disorder is a complicated matter, which translates into difficulty in making this diagnosis and reporting its prevalence. Studies from neurological settings have reported that up to one in five patients can have symptoms not attributed to neurological disease, though prevalence rates of conversion disorder vary by study [4]. The treatment of conversion disorder is no less difficult, especially as it often begins before its etiology is fully understood. Patients with conversion disorder are more likely to have certain psychiatric disorders (e.g., anxiety, depression, and personality disorders) than patients with known neurological disorders, so the traditional dichotomy between psychiatric and neurological conditions is often challenged. Similarly, other functional neurological disorders such as psychogenic non-epileptic seizures, or pseudoseizures, are often present in patients with epileptic seizures, complicating diagnosis and treatment. As such, an approach that aims to treat functional symptoms both physically and psychologically may be the answer. In this case report, we present the background, history, evaluation, and treatment of a complex case of conversion disorder characterized by multiple neurological, dissociative, and amnestic symptoms in a patient with a mild traumatic brain injury and a history of social anxiety.

## Case presentation

Patient background

The patient was a 17-year-old Caucasian female of normal height and weight. She met developmental milestones appropriately. There was no history of separation anxiety, social difficulties, obsessive or repetitive play, significant mood disturbances, or academic concerns during childhood. She reported that her childhood was "normal", except for two moves in the fourth and sixth grades. The adjustment to her new home and school was somewhat difficult, as she missed her old friends. At age 11, she fractured her wrist when she slipped and fell. At age 12, she fractured her leg while roller skating. Around this time, her uncle committed suicide. She had been close to the uncle, and though she had always been described as quiet and soft-spoken, she was noted to have become more socially withdrawn after his passing. Throughout middle school, she developed social anxiety. In the ninth grade, she transferred to an online school due to her social anxiety as well as bullying that occurred after she reported a classmate for suicidal ideation. By this time, the COVID-19 pandemic had already prompted the transition of many schools to an online format, though the patient and her family had already been planning this transition prior to the pandemic. At online school, she was getting above-average grades and was even taking some college-level courses. At the age of 16, she started seeing a therapist every two weeks for her social anxiety, which helped. She was able to keep a job at a grocery store that required interaction with others. She still did not enjoy social activities such as going to parties or other events, but she was doing better. At this time, she also started seeing a psychiatrist, who diagnosed her with social anxiety disorder. She was prescribed fluoxetine starting at 10 mg daily, which was increased to 30 mg daily over the course of a few months. She found that the medication was helpful. Besides her social anxiety disorder, she did not meet the criteria for any other psychiatric disorder and had no history of past psychiatric hospitalization, self-harm, substance use, legal history, or abuse. Her past psychiatric history consisted of evaluation and treatment solely on an outpatient basis.

The patient also had a rather unusual medical history. She was diagnosed with bilateral juvenile cataracts at age 11, red-green color blindness, and new-onset visual snow at age 14. For visual snow, she was prescribed topiramate up to 100 mg nightly without benefit, so the medication was discontinued after several months. As part of the workup of her visual symptoms, she had a brain MRI, which was normal. Due to her younger sister's diagnosis of focal epilepsy, she had a routine electroencephalogram (EEG) at age 15, which was normal. Around that time, her doctor noticed a right carotid bruit and a left-sided murmur with a mid-systolic click. She had an ultrasound, which found no stenosis of the carotid artery, and an echocardiogram, which found trivial mitral insufficiency.

At the time of the following case, the patient lived with her mother (age 38), father (age 42), two sisters (ages 18 and 14), brother (age 12), and four cats. Her family had a notable history of psychiatric and medical conditions. Her mother had a history of depression, anxiety, migraines, polymyositis, and anti-synthetase syndrome. Her father had a history of depression. Her older sister had a history of migraines. Her younger sister had a history of attention-deficit/hyperactivity disorder, tics, focal epilepsy, and migraines. Her brother had a history of autism. There was no strong indication of a dysfunctional family dynamic or emotional neglect felt by the patient, as she reported a good relationship with her family. Her mother, in particular, seemed invested in her care.

Timeline of events

While working at the supermarket one morning, the patient was found lying on her back in the walk-in freezer and was noted to have a small laceration and hematoma on the back of her head. It took about five minutes for her to open her eyes once she was found by coworkers. Reports vary as to whether she was unconscious or simply dazed. She presented to the emergency department (ED) with headache, nausea, unsteady gait, generalized motor weakness, slow speech, flat affect, and amnesia. Her physical exam was otherwise unrevealing, and her neurological exam was non-focal. It was noted that her generalized weakness during the exam was likely limited by effort. As shown in Table 1, serological studies, CT of the head and neck, MRI of the brain and total spine, and a 24-hour EEG were all unrevealing.

**Table 1:** The initial test results of the patient CO_2_: carbon dioxide; BUN: blood urea nitrogen; AST: aspartate aminotransferase; ALT: alanine aminotransferase; PT: prothrombin time; INR: international normalized ratio; WBC: white blood cell; RBC: red blood cell; MCV: mean corpuscular volume; MCH: mean corpuscular hemoglobin; MCHC: mean corpuscular hemoglobin concentration; RDW: red cell distribution width; MPV: mean platelet volume; TSH: thyroid-stimulating hormone; ESR: erythrocyte sedimentation rate; CRP: C-reactive protein; CK: creatine kinase; LDL: low-density lipoprotein; HDL: high-density lipoprotein; hCG: human chorionic gonadotropin; PCP: phencyclidine; THC: tetrahydrocannabinol; PCR: polymerase chain reaction; RSV: respiratory syncytial virus; COVID: coronavirus disease; ECG: electrocardiogram; CT: computed tomography; MRI: magnetic resonance imaging; EEG: electroencephalogram

Test Name	Result	Reference Range
Sodium	136 mmol/L	136-145 mmol/L
Potassium	4.6 mmol/L	3.5-5.1 mmol/L
Chloride	104 mmol/L	98-107 mmol/L
CO_2_	23.0 mmol/L	21.0-31.0 mmol/L
Anion Gap	9 mmol/L	5-12 mmol/L
BUN	18 mg/dL	7-25 mg/dL
Creatinine	1.15 mg/dL	0.60-1.30 mg/dL
Glucose	83 mg/dL	70-99 mg/dL
Calcium	10.3 mg/dL	8.6-10.3 mg/dL
Alkaline Phosphatase	68 IU/L	34-104 IU/L
Albumin	5.4 g/dL	3.5-5.7 g/dL
Total Protein	8.9 g/dL	6.4-8.9 g/dL
AST	22 IU/L	13-39 IU/L
ALT	20 IU/L	7-52 IU/L
Total Bilirubin	2.1 mg/dL	0.3-1.0 mg/dL
PT	13.7 sec	12.0-14.6 sec
PT/INR	1.0	0.8-1.1
WBC	11.5 x 10^3/µL	4.8-10.8 x 10^3/µL
RBC	4.83 x 10^6/µL	4.50-6.10 x 10^6/µL
Hemoglobin	14.6 g/dL	14.0-17.5 g/dL
Hematocrit	44%	39-53%
MCV	91 fL	80-99 fL
MCH	30.3 pg	27.0-34.0 pg
MCHC	33.3 g/dL	31.0-37.0 g/dL
RDW	13.3%	11.0-16.0%
Platelets	326 x 10^3/µL	130-400 x 10^3/µL
MPV	7.4 fL	8.0-13.0 fL
Neutrophil Number	10.20 x 10^3/µL	2.00-8.00 x 10^3/µL
Neutrophil Percent	89%	45-75%
Lymphocyte Number	0.90 x 10^3/µL	0.70-5.20 x 10^3/µL
Lymphocyte Percent	8%	19-46%
Monocyte Number	0.40 x 10^3/µL	0.10-1.30 x 10^3/µL
Monocyte Percent	3%	2-12%
Eosinophil Number	0.20 x 10^3/µL	0.04-0.54 x 10^3/µL
Eosinophil Percent	1%	0-4%
Basophil Number	0.00 x 10^3/µL	0.00-0.21 x 10^3/µL
Basophil Percent	0%	0-1%
TSH	2.24 mU/L	0.4-4.0 mU/L
Vitamin B12	908 pg/mL	160-950 pg/mL
1,25-Dihydroxyvitamin D	47 pg/mL	18-78 pg/mL
Acetaminophen	<10 μg/mL	10-25 μg/mL
Salicylates	<4 mg/dL	4-7 mg/dL
Ethanol	<10 mg/dL	<10 mg/dL
ESR	15 mm/hr	0-20 mm/hr
CRP	6.1 mg/L	0-10 mg/L
Lactic Acid	1.3 mmol/L	0.6-1.4 mmol/L
CK	93 IU/L	30-223 IU/L
Troponin	<0.03 ng/mL	<0.06 ng/mL
Lipase	36 U/L	11-68 U/L
Total Cholesterol	106 mg/dL	<200 mg/dL
Triglycerides	72 mg/dL	<150 mg/dL
LDL Cholesterol	46 mg/dL	<100 mg/dL
HDL Cholesterol	46 mg/dL	<60 mg/dL
Serum Beta-hCG	<1 mIU/mL	<5 mIU/mL
Urine Color	Yellow	-
Urine Appearance	Clear	-
Urine pH	7.5	5.0-8.0
Urine Specific Gravity	1.015	1.005-1.028
Urine Glucose	Negative	-
Urine Ketones	Negative	-
Urine Bilirubin	Negative	-
Urine Blood	Negative	-
Urine Protein	Negative	-
Urine Leukocyte Esterase	Negative	-
Urine Nitrite	Negative	-
Urine Opiate Screen	Negative	-
Urine Amphetamine Screen	Negative	-
Urine Methadone Screen	Negative	-
Urine Cocaine Screen	Negative	-
Urine Barbiturates Screen	Negative	-
Urine Benzodiazepine Screen	Negative	-
Urine PCP Screen	Negative	-
Urine THC Screen	Negative	-
Urine Oxycodone Screen	Negative	-
Urine Fentanyl Screen	Negative	-
Influenza A PCR	Negative	-
Influenza B PCR	Negative	-
RSV PCR	Negative	-
COVID-19 PCR	Positive	-
ECG 12-Lead	Rate 124 bpm, QRS 88 ms, QTC 471 ms	-
CT Brain	Normal	-
CT Cervical Spine	Normal	-
CT Chest	Normal	-
MRI Brain	Normal	-
MRI Cervical Spine	Normal	-
MRI Thoracic Spine	Normal	-
MRI Lumbar Spine	Normal	-
24-hour EEG	No epileptiform activity identified	-

The patient was found to be COVID-19 positive but did not have upper respiratory symptoms.

While in the ED, the patient could not recall events surrounding her fall, events occurring in the ED, as well as some long-term memories. She commented, feeling like she was "in a dream" and could not remember basic facts about herself or her family. She would seemingly not understand what was being said to her and had difficulty with cognitive assessments. She was admitted to the hospital, and over the course of a few days, her motor symptoms, speech, affect, and memory improved. Her altered mental status was deemed to be secondary to a mild traumatic brain injury (concussion) with dissociation. Psychiatry was involved, and she received a benzodiazepine trial, which mildly improved her symptoms. She was discharged home with a walker and followed up with her psychiatrist, neurologist, and a concussion clinic for physical therapy.

Her physical symptoms improved with time. She was noted by her neurologist during a follow-up visit to have full motor strength and balance but was afraid of falling. She was able to walk short distances while reassuring herself by staying near furniture or the wall but preferred to use a walker. One month after the event, she no longer relied on a walker. During further follow-up visits, her neurologist noted that her affect became more engaged, she responded appropriately to questions and commands, and she no longer misunderstood components of the exam, but continued to have some lapses in short- and long-term memory.

The patient continued to have waxing and waning dissociative symptoms in the following weeks. Her mother commented that when her symptoms worsened, she would speak with a flat tone and be unable to answer basic questions, forget how to walk or perform activities of daily living (ADLs), forget what she was doing at the time, get upset with minor things, have mood swings, and at times become paranoid. She did not seem to have an appropriate grasp of reality (e.g., she thought a picture of a cat was a real cat) and had altered sensorium, particularly in relation to pain, reporting hyperesthesia of her bilateral arms and legs, but did not seem to understand the concept of pain when it was explained to her during testing. Her symptoms were largely inconsistent in nature, as at times she would talk in a normal tone, laugh, and even initiate conversations. The only consistent symptom seemed to be her retrograde and anterograde amnesia.

During this time, she began to develop new symptoms, including verbal and motor tics, which progressed from eye twitching to facial and neck twitching, smacking her legs, and repeatedly saying certain phrases such as "happy birthday" and quotes from her favorite TV show, *SpongeBob SquarePants*. Her tics improved after she was prescribed clonazepam 0.5 mg daily by her neurologist, though she continued to report bilateral hand and leg paresthesia as well as reduced perception of pain, heat, hunger, and thirst. At one point, she developed excessive thirst and was drinking at least 2 gallons of water per day and urinating every 30 minutes, but this seemed to resolve on its own. Testing at this time revealed a low urine specific gravity but normal serum electrolytes. It was also noted that she gained approximately 20 pounds from six months prior. Her mother reported that the patient could not tell when she was full and would end up overeating. She also developed a tendency to request the same foods (e.g., cereal, bananas, pancakes, noodles, and candy), watch *SpongeBob SquarePants*, and wear the same headphones all the time. At this time, her follow-up appointments with the concussion clinic were put on hold as her additional symptoms kept evolving and were felt to be unrelated to her concussion.

Three months after the initial event, the patient was found on the floor of her room by her mother, who noted a small abrasion and contusion on her forehead. She again presented to the ED with headache, nausea, and a similar mental status as after her previous concussion. She was verbally responsive to questions as she had been previously, but had difficulty following cognitive assessments and continued to have amnesia. She had no new symptoms. Serological studies, CT and MRI of the brain and cervical spine, and a 72-hour video EEG were all unrevealing. As shown in Table 2, a more extensive workup at this time included electromyography (EMG), serological, and cerebrospinal fluid (CSF) studies with anti-N-methyl-d-aspartate (NMDA) and an autoimmune encephalopathy panel, which were also unrevealing.

**Table 2:** Additional test results of the patient ANA: antinuclear antibody; EMG: electromyography; NMDAR: N-methyl-D-aspartate receptor; CSF: cerebrospinal fluid; WBC: white blood cell; RBC: red blood cell; OCBs: oligoclonal bands; IgG: Immunoglobulin G

Test Name	Result	Reference Range
ANA Titer	1:40, Homogenous	-
EMG, bilateral arms	Normal	-
Serum Autoimmune Encephalopathy Panel	Negative	-
Serum Anti-NMDAR	Negative	-
Serum Lactate/Pyruvate	Normal	-
Plasma Amino Acid Profile, Quantitative	Normal	-
Plasma Acylcarnitine Profile, Quantitative	Normal	-
CSF Color	Colorless	-
CSF Appearance	Clear	-
CSF WBC	0 cells/mm^3	<5 cells/mm^3
CSF RBC	0 cells/mm^3	<1 cells/mm^3
CSF Protein	17 mg/dL	15-60 mg/dL
CSF Glucose	54 mg/dL	50–75 mg/dL
CSF OCBs	Negative	-
CSF IgG Index	0.45 mg/dL	0.00-4.50 mg/dL
CSF Autoimmune Encephalopathy Panel	Negative	-
CSF Anti-NMDAR	Negative	-
CSF Lyme Antibody	Negative	-
CSF Amino Acid Profile, Quantitative	Normal	-
CSF Lactate/Pyruvate	Normal	-

She did have an isolated finding of an elevated ANA titer of 1:40 in a homogenous pattern, which was thought to be incidental.

During a follow-up visit after this event, her neurologist reported no focal neurological findings. The patient was able to stand and walk independently and followed commands, but at this time she was nonverbal, with a blank expression on her face. The neurologist reported that the patient held her arms outstretched when they were left in that position but dropped them when they were bent at the elbows. When the patient saw her psychiatrist, she was recommended to go to the ED for an evaluation of possible catatonia. Despite her mother reporting to have recently seen multiple symptoms of catatonia, including immobility, mutism, staring, echolalia, stereotypy, mannerisms, intermittent excitement, and agitation, the patient did not have symptoms of catatonia during her evaluation in the ED. Nevertheless, she was started on a benzodiazepine trial.

During the psychiatric interview in the ED, the patient and her mother were initially both present in the room. When asked most questions regarding her history, the patient would turn to her mother because she had no memory of past events and looked for confirmation and reassurance. She continued to have short- and long-term amnesia, for example, not knowing what she had just eaten for breakfast and not being able to say where she lived, the names of her family members, or what life was like at home. When asked how the patient was able to function at home, her mother showed a journal with notes and photos that the patient kept on her phone to remind herself who she was, who her family members were, and step-by-step instructions on how to perform ADLs, her daily routines, and how to navigate around the house. Notably, while the patient needed reminders and prompting to complete many procedural tasks, she never lost the ability to use her phone and was playing games on it while her mother was speaking. When her mother was not present and the patient had to speak for herself, she showed little to no distress about her symptoms. When asked directly about her feelings, she stated calmly that it would be easier if she remembered things but denied feelings of depression, anhedonia, or guilt regarding her condition.

Given that an extensive medical and neurological workup had already been performed with unrevealing findings and that a psychiatric etiology had not been as heavily considered, the patient underwent further observation in the inpatient psychiatric setting to better characterize her symptoms and how to treat them. The patient stayed at the psychiatric unit for 10 days. At first, her benzodiazepine trial was continued, but it did not seem to have any significant effect, so it was discontinued after a few days. Symptoms of catatonia were not observed, nor were any tics, which had been the reason clonazepam was originally prescribed by her neurologist a few months prior. Over the course of inpatient hospitalization, the treatment team reported a significant improvement in the patient’s cognitive function, with regression in the presence of her mother. She continued to need reminders of how to complete her ADLs if she was without her journal and doubted her memory recall. The patient and her mother were encouraged to reduce the use of external memory aids and to challenge the patient to remember things on her own. This proved to be helpful, as the patient practiced how to trust her own judgment. After her inpatient hospitalization, the patient was stepped down to a partial hospitalization program, where she continued to work on these skills for several weeks. By this time, the patient had an apparently full recovery of functioning memory, no longer relied on memory aids, and no longer showed regression in the presence of her mother. At times, she would preface her memory recall with statements such as "I think?" or "I guess?" and express a lack of awareness of things like where her seat was, though she would still go to the correct spot when asked. By the end of her partial hospitalization, the patient felt that she had improved enough that she planned to go back to school soon.

## Discussion

To summarize the case, the patient was a 17-year-old female with a history of social anxiety that had improved after going to therapy and taking fluoxetine, which was titrated to 30 mg daily. She had been doing well for herself, going to school, and keeping a job until she had an unwitnessed fall, after which she developed a variety of symptoms, most notably amnesia, but also ambulatory dysfunction, paresthesia, polydipsia, motor and vocal tics, and mood swings, all of which seemed to appear and disappear with no discernible pattern. Her amnestic symptoms were particularly odd and inconsistent. Some symptoms, such as losing procedural memories of ADLs, were suggestive of severe impairments, yet other procedural memories, such as how to use a smartphone, were intact. Similarly, explicit memories at times seemed grossly impaired (e.g., she would state that she did not know the names of family members, where she lived, or anything about her home life), yet elements seemed to be intact, as she described a method to circumvent her impairments by looking at pictures and instructions on her phone. For example, when asked how she knew the adults in her home were her parents, she described how she had pictures of them with herself in various stages of childhood and that she could see herself growing up in those pictures. While a seemingly reasonable way of identifying her parents, the fact that she could remember the details of this procedure while claiming to have no memory of even basic information such as where the bathrooms in her home were is rather remarkable. While it is certainly possible to have acute impairments in explicit and implicit memory, such changes would likely be caused by a severe traumatic brain injury, stroke, seizure, or other neurological insult. If that were the case, the memory impairments would be profound and consistent, and their cause would readily be found by diagnostic brain studies [8]. However, none of this was the case with this patient, suggesting a psychogenic origin for her symptoms.

The most likely diagnosis for this patient is a conversion disorder. The way her symptoms appeared and rapidly varied over several months is inconsistent with any known medical or neurological condition. She had an extensive workup of repeated studies, including serological and CSF studies, brain and spinal imaging, EEG, and EMG, which appears to have effectively ruled out infectious, structural, or demyelinating conditions. She did have a family history of epilepsy, but she never witnessed or reported seizure-like activity, so this was deemed less likely to have been a contributing factor. It is worth noting that she did have two concussions, presenting to the ED both times with acute, post-concussive symptoms that seemed to resolve but left behind other continually evolving symptoms that were not felt to be directly caused by the concussion. These concussions may instead have been triggers for the development of conversion symptoms. According to psychoanalytic theory, conversion disorder is caused by the repression of an unconscious conflict and the conversion of stress into a physical symptom to cope with the conflict. With this patient, it is still uncertain what specific stressors she may have been unconsciously attempting to avoid. She did have several notable childhood stressors, including two physically traumatic falls and the death of her uncle, after which her social anxiety seemed to develop. The extent to which these events have been associated with each other and with feelings of anxiety, fear, or loss through some form of implicit conditioning can only be speculated. It is possible that the unconscious, unresolved conflict that had been created by these past events was exposed by acute re-traumatization in the form of her concussions and the accidental falls assumed to have caused them. The production of her particular symptoms may, in turn, have had a symbolic relation to this unconscious conflict. Perhaps her dissociative amnesia and needing to rely on her mother to care for all her needs represented a regression to an earlier, child-like state, as she was otherwise unable to cope with the overwhelming, traumatic stress that she was confronted with.

Multiple doctors who met the patient during her ED visits and hospitalizations noted that she exhibited dissociative symptoms, seemed to lack an appropriate grasp of reality, and was out of tune with her bodily perceptions. The patient herself mentioned feeling like she was in a dream-like state during her initial presentation to the ED. It has been suggested that symptoms of dissociation, and likewise conversion disorder, are related to trauma and are predominantly psychological phenomena. However, increasing data implicates biological and neuropsychological factors in the development of these symptoms. Brain imaging studies have suggested impaired hemispheric communication as a cause of conversion disorder, and it is believed that excessive cortical arousal can induce negative feedback loops between the cortex and the reticular formation of the brainstem [9]. Increased corticoefferent output, in turn, can inhibit awareness of bodily sensation, which may explain the reported sensory deficits in some patients with conversion disorder. In the case of this patient, her perceptual disturbances as well as her dissociative amnesia would suggest a functional disconnect between the cortical, limbic, and subcortical areas responsible for retrieval of memory, likely exacerbated by her traumatic brain injuries.

When trying to explain what happened, the patient's conscious production of symptoms cannot be completely ruled out. Given her prior social anxiety, it is possible that her production of symptoms was a means by which she escaped the social interactions and obligations of school and work, though there was no proof of this being the case. Regardless, it has been suggested that most patients with conversion disorder experience some degree of secondary gain, conscious or unconscious [10]. In the case of this patient, she could have found comfort in being cared for and being freed from personal obligations immediately following her concussions, and thus unconsciously produced new and evolving symptoms as her initial post-concussive symptoms resolved. Alternatively, or in addition to this, the patient may have felt a degree of emotional neglect as she lived in a household with multiple siblings having other medical and psychiatric conditions possibly requiring increased parental attention, and so her production of symptoms could have been a result of some dysfunctional family dynamic.

In any case, patients with conversion disorder often have a pre-existing psychiatric condition. This patient did have pre-existing anxiety that likely affected the evolution of her symptoms in some way. It is worth noting that during her interactions with doctors and therapists, she appeared to have a pathological doubt that may have presented itself as amnesia. For example, when she was left without her journal, which she had been using as a memory aid, she would be in distress because she did not know what to do next or how to do it. When told to go without the journal, however, she made correct decisions, often without even a single mistake. For example, she would be able to find the bathroom on her own or perform ADLs without issue. Pathological doubt is a symptom that often occurs with obsessive-compulsive disorder but can also be associated with other anxiety disorders [11]. It is certainly possible that the patient simply lacked confidence in her memory recall due to pathological doubt, which would serve as a convenient explanation of amnesia in the context of her conversion disorder.

As the diagnosis of this patient was thought to be a conversion disorder, the treatment of her condition involved a thorough examination of her past medical, psychiatric, and social history, as detailed above. With this examination, however, there was still no definitive explanation for her symptoms. Nevertheless, she and her family were reassured that her symptoms were very real but potentially reversible with proper engagement in a goal-oriented treatment program. While empirical data guiding the treatment of conversion disorder is lacking, a variety of techniques ranging from cognitive-behavioral therapy, insight-oriented psychotherapy, hypnosis, biofeedback, and relaxation training have been effectively employed [12,13]. Treatment should address relevant risk factors, such as comorbid psychiatric conditions, and then focus on recognizing triggering events and minimizing any perpetuating factors. If the patient is a child, it is especially important that their family understands the role of these factors so as not to inadvertently support any continued secondary gain or exacerbation of symptoms. In addition, family members should be mindful of how their attitudes and interactions with the patient may affect them. With this patient, her primary concern was her amnestic symptoms, which she was attempting to treat but also potentially perpetuating by using a journal to remind her of all the information she could not recall. By weaning her off the use of this journal, she demonstrated that her memory functions were intact but that she doubted her recall of even the most basic information. It was through this intervention, as well as continued treatment and follow-up in the inpatient and outpatient settings, that her symptoms improved. In her case, as with many cases of conversion disorder, the importance of a timely, practical, and multisystem approach cannot be understated. In general, the resolution of conversion disorder symptoms is usually spontaneous, but it is known that the longer the duration and progression of symptoms, the more difficult the treatment can become. An approach that uses a thorough examination of the patient and their history to guide an individualized treatment plan addressing all relevant psychological factors, both precipitating and perpetuating, as well as any comorbid conditions, should be initiated as soon as the diagnosis is made.

## Conclusions

Conversion disorder is a complicated condition with a widely variable etiology, presentation, and treatment. It challenges the traditional dichotomy between psychiatric and neurological illnesses, and as such, the approach toward its treatment benefits from the identification of all possible contributing factors. This case report demonstrates the interplay between the biopsychosocial underpinnings of conversion disorder complicated by a traumatic brain injury and acute psychological re-traumatization, focusing on the connection between symptoms of conversion and dissociation with trauma and memory. The neurological, dissociative, and amnestic symptoms seen in this case were addressed through a practical approach with close, continued follow-up. For cases such as this one in which the patient is a child or an adolescent, family members should be mindful of their attitude and interactions with the patient, as well as the role of any other relevant psychological factors, so as to be supportive of the treatment process. The identification of these factors is crucial to the evaluation and treatment of all psychiatric phenomena, and in this case, it helped to show a unique presentation of conversion disorder. Psychiatric diagnoses are only imperfect attempts at capturing the intricacies of reality with the purpose of organizing our thinking to communicate the needs of the patient, so care must be given to fully understand the patient and their condition so that appropriate and effective treatment may be provided.
